# Simultaneous Epstein Barr Virus and Cytomegalovirus Infection Accompanied by Leiomyomatous Change in a Well-Differentiated Liposarcoma in a Patient With Long-Term Corticosteroid Treatment

**DOI:** 10.1080/13577149778489

**Published:** 1997-03

**Authors:** Paul R. A. Sars, Willemina M. Molenaar, Jan Koudstaal, Harald J. Hoekstra

**Affiliations:** 1 Department of Surgical Oncology Groningen University Hospital The Netherlands; 2 Department of Pathology Groningen University Hospital The Netherlands; 3 Department of Pathology University Hospital Hanzeplein 1, PO Box 30.001 Groningen NL-9700 RB The Netherlands

## Abstract

*Patient*. A 59-year-old woman presented with a large tumour of the abdominal wall. She had been taking corticosteroids for severe chronic obstructive pulmonary disease for 15 years. On CT scan the tumour had the characteristics of lipomatous tissue with a dense core.

*Results*. Histology showed a well-differentiated liposarcoma with a core of benign fibroleiomyomatous differentiation. Within the core, a third component was observed, characterized by more pleomorphism and the presence of an inflammatory infiltrate. In this component, immunoperoxidase stains and *in situ* hybridization demonstrated cytomegalovirus (CMV) and Epstein Barr virus (EBV) infection in large and small cells, respectively.

*Discussion*. Long-term corticosteroid use for pulmonary disease may extend the list of immunosuppressed states associated with the development of leiomyomatous tumours with EBV infection, previously described in AIDS patients and liver transplant recipients. The role of CMV is uncertain.


*Sarcoma* (1997) 1, 55–58

CASE REPORT

### Simultaneous Epstein Barr virus and cytomegalovirus infection accompanied by leiomyomatous change in a well-differentiated liposarcoma in a patient with long-term corticosteroid treatment

PAUL R. A. SARS,[1] WILLEMINA M. MOLENAAR,[2] JAN KOUDSTAAL[2] & HARALD J. HOEKSTRA[1]

*Departments of [1]Surgical Oncology and [2]Pathology, Groningen University Hospital, The Netherlands*

**Abstract**

*Patient.* A 59-year-old woman presented with a large tumour of the abdominal wall. She had been taking corticosteroids for severe chronic obstructive pulmonary disease for 15 years. On CT scan the tumour had the characteristics of lipomatous tissue with a dense core.

*Results.* Histology showed a well-differentiated liposarcoma with a core of benign fibroleiomyomatous differentiation. Within the core, a third component was observed, characterized by more pleomorphism and the presence of an inflammatory infiltrate. In this component, immunoperoxidase stains and *in situ* hybridization demonstrated cytomegalovirus (CMV) and Epstein Barr virus (EBV) infection in large and small cells, respectively.

*Discussion.* Long-term corticosteroid use for pulmonary disease may extend the list of immunosuppressed states associated with the development of leiomyomatous tumours with EBV infection, previously described in AIDS patients and liver transplant recipients. The role of CMV is uncertain.

**Key words**: *liposarcoma, aetiology, corticosteroids, cytomegalovirus, Epstein Barr virus.*

### Introduction

Liposarcomas constitute 16–18% of the soft tissue sarcomas with a peak incidence between the ages of 40 and 60 years. There are four major types of liposarcoma: myxoid liposarcoma, round cell liposarcoma, well-differentiated liposarcoma and pleomorphic liposarcoma.[1] The well-differentiated subtype of liposarcoma may show dedifferentiation resulting in a high-grade sarcoma often showing a morphology resembling malignant fibrous histiocytoma.[2,3] Heterologous differentiation within the dedifferentiated component towards skeletal muscle, smooth muscle, bone and osteoid or vessels has previously been described. These dedifferentiated components generally show malignant features, but may also appear benign, especially in the case of smooth muscle differentiation.[2–8]

The aetiology of soft tissue sarcomas is still unknown. Sarcomas may develop in scar tissue, especially after burns, and after intensive radiotherapy.[1,9] Exposure to chemical products, such as dioxin and herbicides, might play a role as well.[10,11] Recently, there have been reports suggesting a relation between Epstein Barr virus (EBV) infection and the development of smooth muscle tumours in patients with AIDS and liver transplant recipients.[12–16]

We report a case of a patient with a well-differentiated liposarcoma which showed central leiomyomatous change concurrent with both EBV and cytomegalovirus (CMV) infection.

### Case history

A 59-year-old Caucasian female was admitted to another hospital because of an aggravation of uremia and asthma not responding to oral steroids and antibiotics. She had suffered from chronic obstructive pulmonary disease for over 40 years for which she had used steroids orally for 15 years and an inhaler for more than a decade; the daily steroid dosage depended on the patient's pulmonary status. Previous history further revealed hypertension, gastric ulcer and thyroidectomy because of a multinodular goitre. Upon routine physical examination, there was wheezing over both lungs and a large mass was found in the right lower abdomen. She was treated for her pulmonary complaints with high

Correspondence to: W. M. Molenaar, Department of Pathology, University Hospital, Hanzeplein 1, PO Box 30.001, NL-9700 RB Groningen, The Netherlands. Tel: +31 50 361 4244/4684; Fax: +31 50 363 2510; E-mail: jacobs@postbus.rug.nl.

1357-714X/97/010055-04 ©1997 Journals Oxford Ltd

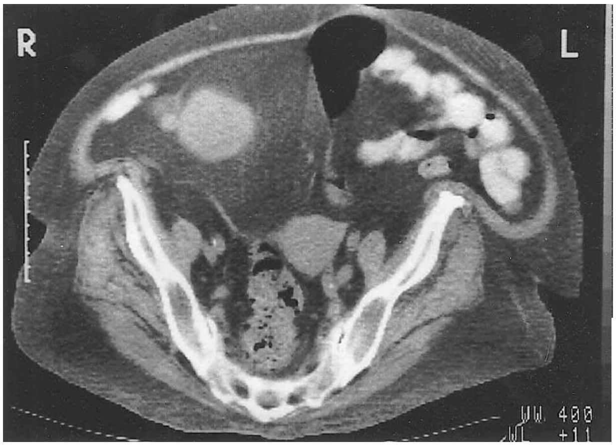

**Fig. 1.** *CT scan showing a lipomatous tumour originating from the abdominal wall with a central denser component.*

doses of intravenous steroids, ipratropium, fenoterol, theophylline and antibiotics. After her pulmonary condition improved, a CT scan was performed to evaluate the abdominal mass. This showed a tumour of the abdominal wall with a diameter of 18 cm with the density of lipomatous tissue in the periphery and a denser core (Fig. 1).

The patient was referred to the department of surgical oncology of our hospital for further evaluation of the tumour. A tru-cut biopsy showed a mesenchymal tumour, which was difficult to classify. Shortly after this procedure, her condition deteriorated and she developed severe dyspnoea and acute heart failure with anuria. She died one day after transfer to our hospital. A postmortem examination was performed.

### Methods

The tru-cut biopsy and samples from the tumour taken at postmortem were examined on H&E-stained paraffin sections. Standard immunperoxidase stains were performed using antibodies to vimentin, smooth muscle actin and desmin on paraffin and frozen sections. Immunohistological stains for the detection of EBV (LMP, clone CS 1–4; DAKO) and CMV (IEA, clone E13; Argene Biosoft) proteins were performed on paraffin sections and frozen sections after high temperature antigen retrieval, using the multilink-label technology of Biogenex. Final colour was developed by the BCIP/NBT ($\beta$-chloro-indolyl-phosphate/nitro blue tetrazolium) method.

*In situ* hybridization studies were performed on standard paraffin-embedded tissue. Tissue sections (5 $\mu$m) were floated in a bath of distilled water, mounted on 3-aminopropylethoxysilane (APES)-coated slides and heated for 15 min at 60$^{\circ}$C on a hotplate. They were dewaxed in xylene and rehydrated in serial ethanol washes (100%, 95% and 70%), then digested with 20 $\mu$g ml$^{-1}$ of proteinase-K for 60 min at 37$^{\circ}$C. After blocking, rinsing and dehydration the slides were covered by a mixture of either CMV (British Technology) or EBV (EBER; DAKO) specific oligonucleotides. CMV oligonucleotides were labelled with digoxigenin (DIG) and EBV oligonucleotides were labelled with FITC. The slides were heated for 5 min at 92$^{\circ}$C and incubated overnight at 37$^{\circ}$C. Detection was carried out using anti-FITC–alkaline phosphatase for EBV and anti-DIG–alkaline phosphatase for CMV. Final colour was developed by the BCIP method.

### Results

The most significant findings at postmortem were bronchopneumonia in the upper lobes of both lungs and a tumour originating from the lower right abdominal wall without relation to the intra-abdominal organs. The tumour, which had a maximum diameter of 18 cm, had a smooth and lobulated surface and was completely surrounded by a capsule. On cross-section the periphery had the aspect of adipose tissue with a central core of firm white tissue with a diameter of 7 cm. There were no signs of metastases.

Microscopic examination revealed foci of *Aspergillus* infection in both lungs. The alveolar epithelium showed eosinophilic intranuclear inclusions typical of a viral infection (Fig. 2). These cells were immunoreactive for CMV protein and showed a positive signal for the CMV genome with *in situ* hybridization. In addition, immunoreactivity for EBV protein was demonstrated and there was a positive signal for the EBV genome with *in situ* hybridization. The peripheral part of the tumour of the abdominal wall showed adipose tissue with foci of fibrosis (Fig. 3). The fat vacuoles were of different size. Convincing multi-vacuolated tumour cells were not observed and there was no mitotic activity. However, some fat cells showed finely vacuolated cytoplasm and/or hyperchromatic nuclei. These

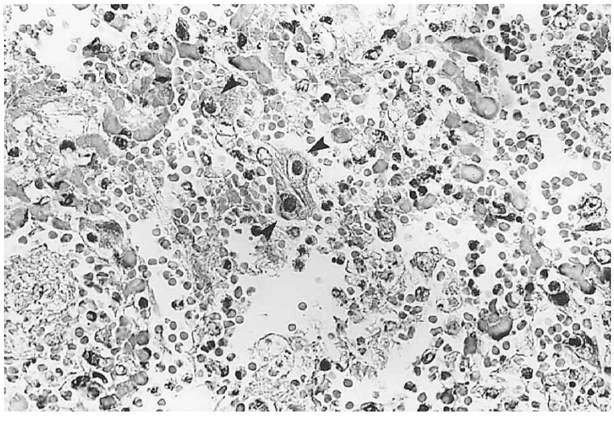

**Fig. 2.** *Alveolar epithelium with eosinophilic inclusions in the nuclei (arrowheads) typical of CMV infection (H&E; ×400).*

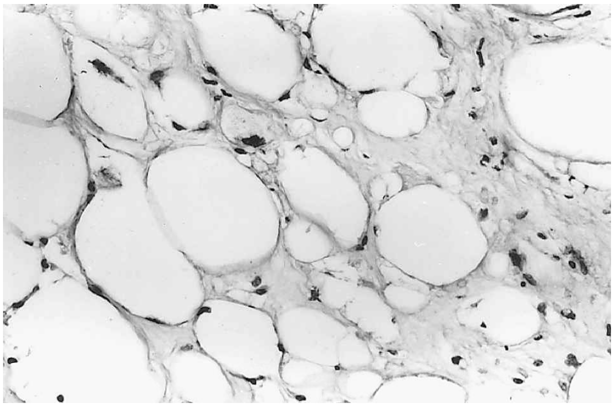

**Fig. 3.** *Peripheral part of the tumour of the abdominal wall, showing well-differentiated liposarcoma (H&E; ×256).*

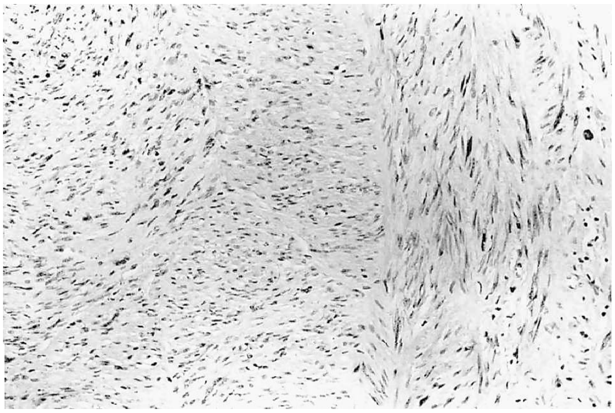

**Fig. 4.** *Central fibroleiomyomatous part of the tumour (H&E; ×160).*

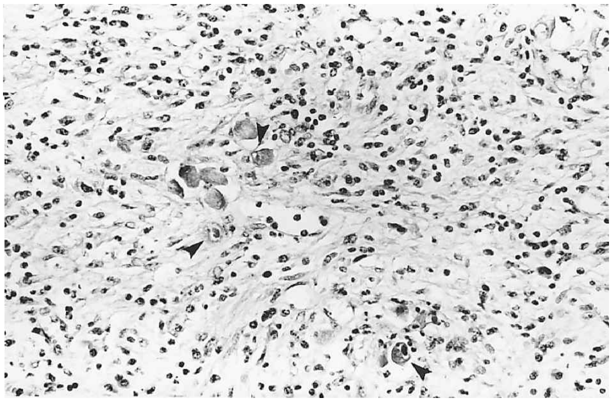

**Fig. 5.** *Central leiomyomatous part of the tumour with nuclear inclusions (arrowheads), suggestive of viral infection and inflammatory infiltrate (i; H&E; ×256).*

morphological features, together with the deep location and the size of the tumour, led to a diagnosis of well-differentiated liposarcoma rather than lipoma. The central part of the tumour was composed of bundles of spindle-shaped cells, some of which displayed hyperchromatic irregular nuclei (Fig. 4); no mitotic activity or necrosis was observed. This part

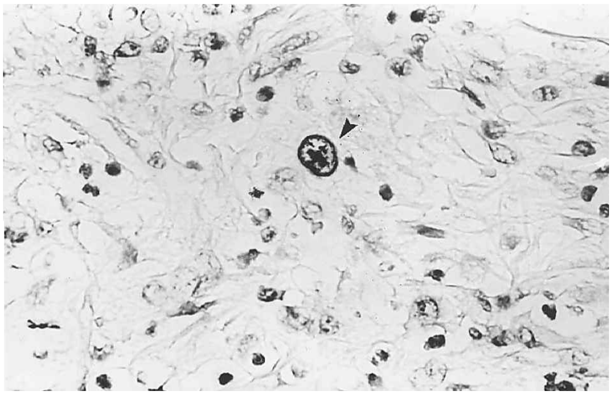

**Fig. 6.** *Immunoperoxidase stain for CMV in the central part of the tumour showing reaction in large nuclei (arrowheads; ×526).*

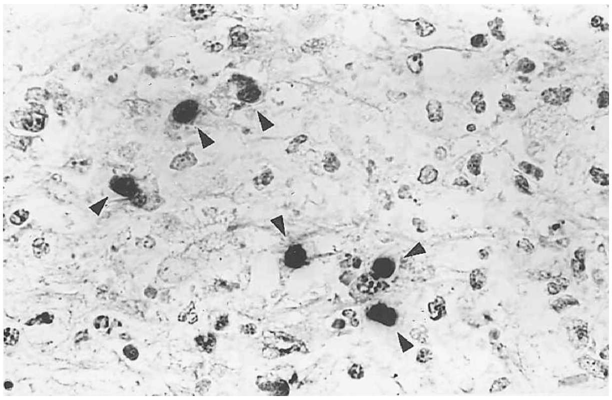

**Fig. 7.** *EBER-1 mRNA in smaller nuclei in the central part of the tumour (arrowheads; ×526).*

of the lesion resembled a fibroleiomyomatous tumour without evidence of malignancy. Immunoperoxidase stains for vimentin, actin and desmin were positive. A third component was present in the central part of the tumour and formed the only constituent of the tru-cut biopsy. The most remarkable feature was the presence of large cells with prominent eosinophilic intranuclear inclusions embedded in a spindle cell background (Fig. 5). A prominent inflammatory infiltrate was present, largely consisting of polymorphonuclear leucocytes, as well as foci of necrosis.

Immunoperoxidase stains for CMV proteins were strongly positive in the large cells (Fig. 6); the immunoperoxidase reaction for LMP-1 EBV was slightly positive in smaller tumour cells. *In situ* hybridization showed a positive signal for the CMV genome in the large cells, which were also CMV protein immunoreactive. EBER-1 mRNA was demonstrated in smaller cells (Fig. 7).

### Discussion

The current report describes a tumour consisting of well-differentiated liposarcoma with a central core of leiomyoma. The latter part appeared to be affected by both a CMV and an EBV infection. Dedifferentiation of well-differentiated liposarcomas is a well-established phenomenon and several reports have described the occurrence of heterologous components such as skeletal muscle, smooth muscle, bone or osteoid, or vessels.[2–8] In most cases, these heterologous components are clearly malignant. In our case the central leiomyomatous component of the tumour showed no signs of malignancy.

Recently, several reports[12–15] have described the occurrence of leiomyomas and leiomyosarcomas in HIV-positive patients and McClain *et al.*[12] have suggested a relation between EBV infection and the development of leiomyomas in these patients. Similar observations have been reported in liver transplant recipients.[16] These findings lead to the suggestion that the leiomyomatous differentiation and the EBV infection in our case of well-differentiated liposarcoma are also related. A parallel between AIDS patients, transplant recipients and our patient is a marked decrease in immunologic surveillance, which in our patient was brought about by the use of steroids for chronic obstructive pulmonary disease for 15 years. This also explains her *Aspergillus* and CMV bronchopneumonia. To the best of our knowledge, a relation between smooth muscle tumours and CMV infection has not yet been described. However, the presence of a CMV infection restricted to the leiomyomatous part of her tumour suggests the possibility of a relation between CMV and the formation of smooth muscle tumours as well.

### References

1 Enzinger FM, Weiss SW. *Soft tissue tumors*, 3rd edn. St Louis: Mosby, 1995.

2 Weiss SW, Rao VK. Well-differentiated liposarcoma (atypical lipoma) of deep soft tissue of the extremities, retroperitoneum, and miscellaneous sites. A follow-up study of 92 cases with analysis of the incidence of dedifferentiation. *Am J Surg Pathol* 1992; 16:1051–8.

3 McCormick D, Mentzel T, Beham A, *et al.* Dedifferentiated liposarcoma. Clinicopathologic analysis of 32 cases suggesting a better prognostic subgroup among pleomorphic sarcomas. *Am J Surg Pathol* 1994; 18:1213–23.

4 Salzano RP, Tomkiewicz Z, Arficano WA. Dedifferentiated liposarcoma with features of rhabdomyosarcoma. *Conn Med* 1991; 55:200–2.

5 Tallini G, Erlandson RA, Brennan MF, *et al.* Divergent myosarcomatous differentiation in retroperitoneal liposarcomas. *Am J Surg Pathol* 1993; 17:546–56.

6 Evans HL, Khurana KK, Kemp BL, *et al.* Heterologous elements in the dedifferentiated component of dedifferentiated liposarcoma. *Am J Surg Pathol* 1994; 18:1150–7.

7 Evans HL. Smooth muscle in atypical lipomatous tumors. A report of three cases. *Am J Surg Pathol* 1990; 14:714–8.

8 Suster S, Wong T-Y, Moran CA. Sarcomas with combined features of liposarcoma and leiomyosarcoma. Study of two cases of an unusual soft-tissue tumor showing dual lineage differentiation. *Am J Surg Pathol* 1993; 17:905–11.

9 Laskin WB, Silverman TA, Enzinger FM. Postradiation soft tissue sarcomas; an analysis of 53 cases. *Cancer* 1988; 62:2330–40.

10 Hardell L, Eriksson M. The association between soft tissue sarcomas and exposure to phenoxyacetic acids. *Cancer* 1988; 62:652–6.

11 Kang H, Enzinger FM, Breslin P, *et al.* Soft tissue sarcoma and military service in Vietnam: a case control study. *J Natl Cancer Inst* 1987; 79: 693–9.

12 McClain KL, Leach CT, Janson HB, *et al.* Association of Epstein-Barr virus with leiomyosarcomas in young people with AIDS. *N Engl J Med* 1995; 332:12–8.

13 Prevot S, Neris J, de Saint Maur PP. Detection of Epstein-Barr virus in an hepatic leiomyomatous neoplasm in an adult human immunodeficiency virus 1-infected patient. *Virch Arch* 1994; 425:321–5.

14 Rabkin CS. Epidemiology of AIDS-related malignancies. *Curr Opin Oncol* 1994; 6:492–6.

15 Dahan H, Beges C, Weiss L, *et al.* Leiomyoma of the adrenal gland in a patient with AIDS. *Abdom Imaging* 1994; 19:259–61.

16 Timmons CF, Dawson DB, Richards CS, *et al.* Epstein-Barr virus-associated leiomyosarcomas in liver transplantation recipients—origin from either donor or recipient tissue. *Cancer* 1995; 76:1481–9.
